# Do minimum acceptable diet and family caregiving mediate the associations of maternal education and household wealth with childhood stunting and wasting in Sri Lanka?

**DOI:** 10.1017/S1368980026101888

**Published:** 2026-01-23

**Authors:** Damith Chandrasenage, Paula Griffiths, William Johnson

**Affiliations:** 1 School of Sport, Exercise and Health Sciences, Loughborough University, Loughborough, UK; 2 Department of Social Statistics, Faculty of Social Sciences, University of Kelaniya, Kelaniya, Sri Lanka; 3 Department of Health Sciences, University of York, York, UK

**Keywords:** Stunting, Wasting, Socio-economic position, Minimum acceptable diet, Family caregiving, Mediation analysis, Sri Lanka

## Abstract

**Objective:**

To investigate the extent to which the associations of socio-economic position (SEP) with stunting and wasting are mediated by minimum acceptable diet (MAD) and a family care indicator (FCI) in Sri Lanka.

**Design:**

Secondary data analysis of children from the 2016 Sri Lanka Demographic and Health Survey. The outcomes were stunting and wasting, the exposure was a composite measure combining maternal education and household wealth, and the mediators were binary MAD and FCI variables (adequate *v*. inadequate). Analyses were performed using counterfactual mediation models adjusted for age, sex and place of residence.

**Setting:**

A nationally representative sample of children from Sri Lanka.

**Participants:**

Mothers/caregivers of children under 36 months (4325).

**Results:**

Twenty per cent of children were stunted, and 14 % were wasted. Lower SEP was associated with higher odds of stunting and wasting and inadequate MAD and FCI. Inadequate FCI was associated with higher odds of stunting (OR = 1·47, 95 % CI = 1·24, 1·74) but not wasting (OR = 1·14, 95 % CI = 0·94, 1·38), whereas MAD was not associated with stunting or wasting. Neither MAD nor FCI significantly mediated the relationship between SEP and stunting and wasting. All mediation estimates were statistically non-significant at the 5 % level. For example, the proportion mediated by FCI on the association between the lowest composite SEP and stunting was 13 % (mean difference = 0·13, 95 % CI = < 0·00, 0·26).

**Conclusion:**

We did not find consistent or strong evidence that the associations of SEP with childhood stunting and wasting in Sri Lanka are mediated by MAD and FCI. Research with larger samples is needed for more precise estimates.

## Key messages

- Levels of childhood stunting and wasting, adequate minimum acceptable diet and family care were strongly associated with socio-economic position.
- Although a large proportion of the associations between maternal education, household wealth, and childhood stunting and wasting were mediated by minimum acceptable diet and family caregiving in some SEP groups, this was not consistent across all groups.
- Further research with larger samples is needed because all estimates of the proportion mediated and natural indirect effect were not statistically significant at α of 5 % with wide CI for the estimates.

The global prevalence of under-5 stunting and wasting is 22·3 % and 6·8 %, respectively. In South Asia, the prevalence of under-5 stunting and wasting is 31·4 % and 14·8 %, respectively^(1)^. While Sri Lanka is performing better in terms of nutrition indicators compared to many other South Asian countries, the prevalence of stunting in 2022 remained unacceptably high at 15·9 %, although this rate is well below the regional average of 31·4 %. Further, the prevalence of wasting in Sri Lanka was 15·1 % in 2016, close to the South Asian regional average of 16·0 % for the same year^(1)^.

As in all low-and middle-income countries, lower socio-economic position (SEP) is associated with greater risks of stunting and wasting in Sri Lanka. Previous studies have reported that lower maternal education and poorer household wealth are strongly associated with high risk of stunting and wasting in Sri Lanka^(2–4)^.

Child nutrition indicators in Sri Lanka are relatively positive^(5)^, with a high exclusive breast-feeding rate of 81 % in 2016, and 62 % of children meeting the minimum acceptable diet (MAD: a binary indicator of feeding practice that assesses the quality and sufficiency of a child’s diet between ages 6 and 23 months)^(6)^. There is limited evidence on the association between MAD and child undernutrition, but existing studies suggest that low MAD is associated with a higher risk of child stunting and wasting in Sri Lanka^(4)^.

Caring practices play a crucial role in determining maternal and child nutrition outcomes, as highlighted by the UNICEF Nurturing Care Framework^(7)^. This framework identifies five key components, good health, adequate nutrition, responsive caregiving, opportunities for early learning, and security and safety, as essential for optimal child development. Responsive caregiving, in particular, directly influences feeding practices, emotional bonding and the overall well-being of both mother and child. Evidence shows that when caregivers are responsive and emotionally adapted, children are more likely to receive adequate and appropriate feeding, leading to better growth and reduced risk of malnutrition^(8)^. These findings underline the importance of integrating nurturing care principles into nutrition interventions to ensure holistic support for mothers and children.

The family care indicator (FCI) captures aspects of the home caregiving environment, including play materials, children’s books and adult–child interactions. It has been used in low-and middle-income countries as a validated proxy for responsive caregiving^(8)^, a domain of the UNICEF Nurturing Care Framework. Although formal validation in Sri Lanka is limited, the FCI provides a meaningful measure of household care quality relevant to child growth and development. There is no existing evidence on the association between child undernutrition and FCI (FCI: a binary indicator of adequate quality household caregiving environment) in Sri Lanka.

Understanding the mechanisms through which SEP affects the risk of childhood stunting and wasting is important in creating effective, targeted, and equitable interventions that can reduce the prevalence and impact of these conditions on children’s health and well-being^(9,10)^. It is possible that MAD and FCI mediate the association of SEP with childhood wasting and stunting in Sri Lanka because (1) there are known socio-economic inequalities in MAD and FCI in Sri Lanka and (2) inadequate MAD and FCI are associated with higher rates of stunting and wasting in Sri Lanka. Studies have investigated this possibility in other settings but found inconsistent results (e.g. of the proportion mediated) likely due to differences in study designs, measures and analyses. In a recent study of 132 448 under-5 children from thirteen countries in West Africa, Dwomoh *et al.* (2022)^(11)^ reported that 35·9 % (95 % CI = 12·4, 59·3) of the association of maternal education with stunting was mediated by MAD. For household wealth, the equivalent estimate was 37 % (95 % CI = 11·5, 61·5). The setting and context in Sri Lanka are, however, very different, and there is no comparable evidence for Sri Lanka.

While previous studies in South Asia have documented socio-economic inequalities in child malnutrition and feeding practices^(12–15)^, none have applied mediation analysis using nationally representative data to explicitly examine how caregiving and diet pathways explain socio-economic disparities in child undernutrition. Our study addresses this gap in the Sri Lankan context. This study aimed to investigate how the associations of maternal education and household wealth with childhood stunting and wasting are mediated by MAD and FCI in Sri Lanka.

## Material and methods

### Surveys

The present study used the latest available 2016 Demographic and Health Surveys (DHS) data of Sri Lanka. The DHS is a nationally representative household survey. As part of the survey, it collects health and demographic data of children under 5 years old by interviewing women of reproductive age (15–49 years). Data were collected by trained enumerators recruited by the Department of Census and Statistics. Enumerators underwent intensive training, including classroom instruction, field practice and standardisation exercises before field deployment. Anthropometric data (height and weight) were collected by trained nursing sisters using SECA digital weighing scales and SECA measuring boards, following WHO protocols. Children under 24 months were measured lying down, while older children were measured standing. Two measurements were taken for each child, with a third if discrepancies exceeded the acceptable range. Supervisors conducted random spot checks to ensure data quality^(6)^. More details about the DHS survey and sampling design can be found on the DHS website (https://dhsprogram.com/). The Sri Lanka DHS 2016 data is not freely available for online download; access requires special permission from the Department of Census and Statistics in Sri Lanka.

### Sample

We used two samples, one for FCI and one for MAD. The FCI sample comprised 4325 children aged 0–35 months. The MAD sample comprised 2190 children aged 6–23 months. We could not include children outside of this age range because MAD is defined based on feeding between 6 and 23 months. All children in the MAD sample were in the FCI sample.

### Outcomes

Child height-for-age and weight-for-height Z-scores were computed according to the WHO-2006 Standards. The outcomes of stunting and wasting were defined as Z-scores more than 2 sd below the median (i.e. 50th percentile).

### Exposure

A composite exposure combining two measures of SEP, maternal education and a wealth index, was considered. Maternal education had two levels: secondary or below education and higher education. Household wealth had three levels: poor, middle and richer. The composite exposure was used to make a meaningful analysis with a combination of the mother’s education and the wealth index minimising the effect of small sample size in subgroups^(16)^. Therefore, the composite exposure had six levels: (1) higher education and richer wealth [reference], (2) higher education and middle wealth, (3) higher education and poorer wealth, (4) secondary or below education and richer wealth, (5) secondary or below education and middle wealth and (6) secondary or below education and poor wealth.

### Potential confounders

Potential confounders included in the analyses were sex of child (male or female), child’s age (months) and resident place (rural or urban).

### Potential mediators

We considered two potential mediators.

MAD is a composite indicator combining minimum dietary diversity (MDD) and minimal meal frequency (MMF). MDD refers to the consumption of foods and beverages from at least five out of eight food groups during the previous day. MMF refers to the consumption of solid, semi-solid or soft foods at least the minimum number of times during the previous day. For breastfed children to have adequate MAD, they need to meet the MDD and MMF requirements. For non-breastfed children to have adequate MAD, they need to meet the MDD requirement (but excluding the dairy products category to avoid double-counting) and the MMF requirement and two or more milk feeds from country-specific infant formula or milk, such as tinned, powdered or fresh animal milk or yogurt^(17)^. MAD is classified as ‘Adequate’ or ‘Inadequate’.

FCI is a composite indicator combining three subgroups: source of play materials, number of books for children and play activities^(8)^. Source of play materials assesses whether the play materials were homemade or from a shop or household objects (1 = one or more sources, 0 = no access). The number of books includes the number of picture or other types of children’s books (1 = one or more books, 0 = no books). The play activities component includes six types of activities done by any adult in the home with the child in the 3 d prior to the survey (1 = one or more activities, 0 = no activities). FCI is classified as ‘Adequate’ if the summation of the three subgroups (source of play materials, number of books for children and play activities) is greater than 1. Otherwise, it is classified as ‘Inadequate’.

### Conceptual and analytical framework

This study was conceptually guided by the UNICEF Nurturing Care Framework, which emphasises nutrition and caregiving as central to child growth and development. In this framework, SEP is hypothesised to influence stunting and wasting both directly and indirectly through caregiving and nutrition-related pathways. In our analysis, we operationalised two domains of the UNICEF Nurturing Care Framework: (i) adequate nutrition, captured by MAD and (ii) responsive caregiving/early learning/safety, captured by FCI.

### Statistical analysis

Descriptive statistics were computed using sampling weights.

Prior to mediation analyses, binary logistic regression models were used to estimate the separate crude associations between the (1) exposure and outcomes, (2) exposure and mediators and (3) mediators and outcomes.

Counterfactual mediation analysis was performed using the G-computation formula in STATA software. Counterfactual mediation models were used to examine the extent to which the association between SEP and child stunting and wasting was mediated by the FCI and, separately, by MAD. Counterfactual mediation allows estimation of direct and indirect effects while appropriately accounting for confounding, non-linearity and interactions. Unlike the traditional approach, it defines mediation in causal terms using potential outcomes, providing more robust and interpretable estimates^(18)^. Four models were developed to decompose the total effects (TE) into natural direct effects (NDE) and natural indirect effects (NIE) and obtain estimates of the percentage mediated:

1. SEP, FCI, stunting.
2. SEP, MAD, stunting.
3. SEP, FCI, wasting.
4. SEP, MAD, wasting.

All models were adjusted for sex, age and place of residence. Following the advice of Alwin and Hauser (1975) and MacKinnon *et al.* (2000)^(19,20)^, the proportion mediated was calculated by dividing the absolute NIE by the sum of the absolute NDE and NIE because suppression (when the TE is less than direct effect) existed in the estimated models. We also report the default proportion mediated values provided by Stata.

## Results

Approximately 20 % of children were stunted and 15 % were wasted; 60 % of children did not have adequate FCI and 39 % did not have adequate MAD (Table 1).

**Table 1:** Descriptive statistics

	FCI sample		MAD sample	
	*n* 4325		*n* 2190	
	Mean	sd	Mean	sd
HAZ	−0·96	1·31	−0·93	1·35
WAZ	−0·78	1·26	−0·73	1·27
	*n*	%	*n*	%
Outcome				
Stunting				
No	3468	80	1765	81
Yes	857	20	425	19
Wasting				
No	3699	86	1872	85
Yes	626	14	318	15
Mediator				
MAD				
Adequate	–		1326	61
Inadequate	–		864	39
FCI				
Adequate	1726	40	–	
Inadequate	2599	60	–	
Exposure				
Mother’s educational				
Higher	2238	52	1171	53
Secondary	2087	48	1019	47
Wealth index				
Rich	1425	33	723	33
Middle	1440	33	747	34
Poor	1460	34	720	33
Composite SEP				
Higher-Rich	1123	26	590	27
Higher-Middle	717	17	371	17
Higher-Poor	398	9	210	10
Secondary or below rich	318	7	142	6
Secondary or below middle	725	17	374	17
Secondary or below-Poor	1044	24	503	23
Covariates				
Sex of child				
Male	2184	51	1098	50
Female	2141	50	1092	50
Age of child (months)				
0–5	520	12	–	
6–23	2190	51	2190	100
24–35	1615	37	–	
Resident place				
Urban	670	15	350	16
Rural	3655	85	1840	84

FCI, family care indicator; MAD, minimum acceptable diet; HAZ, height-for-age z-score; WHZ, weight-for-height z-score; SEP, socio-economic position.

Table 2 shows the descriptive statistics stratified by stunting status and wasting status. The prevalence of stunting and wasting was higher in lower SEP groups. Within each maternal education group, there was a pattern across the household wealth groups. For example, just focusing on the higher maternal education group, the prevalence of stunting was 20 % in the poor wealth group, 17 % in the middle wealth group and 13 % in the rich wealth group (in the FCI sample). Similarly, within each wealth category, there was a pattern of higher rates of stunting in the secondary or below (compared to the higher) maternal education group. In all instances, the prevalence of stunting (or wasting) was lower in the adequate MAD (or FCI) group than in the inadequate MAD (or FCI) group.

**Table 2:** Descriptive statistics, stratified by stunting and wasting status

	FCI sample (*n* 4325)								MAD sample (*n* 2190)							
	Stunting				Wasting				Stunting				Wasting			
	No		Yes		No		Yes		No		Yes		No		Yes	
	*n*	%	*n*	%	*n*	%	*n*	%	*n*	%	*n*	%	*n*	%	*n*	%
Exposure																
Composite SEP																
Higher and rich	981	87	142	13	982	88	131	12	521	88	69	12	519	89	65	11
Higher and middle	593	83	124	17	632	88	90	12	300	81	71	19	328	87	48	13
Higher and poor	318	80	80	20	342	85	61	15	173	82	37	18	178	84	33	16
Secondary or below and rich	264	83	54	17	272	87	40	13	117	83	25	17	119	86	20	14
Secondary or below and middle	565	78	160	22	607	85	111	15	290	77	84	23	314	85	57	15
Secondary or below and poor	747	72	297	28	864	82	193	18	364	73	139	28	414	81	95	19
Mediators																
Minimum acceptable diet																
Adequate	–		–		–		–		701	81	163	19	737	85	127	15
Inadequate	–		–		–		–		1064	80	262	20	1135	86	191	14
Family care indicator																
Adequate	1408	82	318	18	1501	87	225	13	–		–		–		–	
Inadequate	2060	79	539	21	2198	85	401	15	–		–		–		–	

FCI, family care indicator; MAD, minimum acceptable diet; SEP, socio-economic position.

Data are weighted using sampling weights.

### Crude associations

The crude unadjusted associations from binary logistic models are shown in online supplementary material, Supplemental Table S1. Lower SEP was associated with higher odds of stunting and wasting (in both samples) and inadequate FCI and MAD. Inadequate FCI was associated with higher odds of stunting (OR = 1·47, 95 % CI = 1·24, 1·74)) but not wasting (OR = 1·14, 95 % CI = 0·94, 1·38), whereas MAD was not associated with stunting or wasting.

### Mediation results

The proportion mediated by FCI and MAD in each level of the composite SEP variable of mother’s education and household wealth for stunting and wasting is shown, respectively, in Table 3.1 and Table 3.2. The estimates are interpreted as mean difference (MD; the difference in mean potential outcomes under different levels of exposure and/or mediator adjusted for child age, sex and place of residence).

**Table 3.1:** Counterfactual mediation models testing the extent to which the association of socio-economic position with stunting is mediated by family care index and, separately, minimum acceptable diet

	Exposure										
	Higher-Rich (referent)	Higher-Middle		Higher-Poor		Secondary or below rich		Secondary or below middle		Secondary or below-Poor	
		MD	95 % CI	MD	95 % CI	MD	95 % CI	MD	95 % CI	MD	95 % CI
Mediator											
Family care index											
Total causal effect	–	0·05	0·02, 0·09	0·07	0·03, 0·12	0·04	–0·01, 0·09	0·09	0·05, 0·13	0·15	0·12, 0·19
NDE	–	0·05	0·01, 0·08	0·07	0·03, 0·12	0·03	–0·02, 0·08	0·09	0·05, 0·13	0·13	0·10, 0·17
NIE	–	0·01	–0·01, 0·02	< 0·00	–0·02, 0·02	0·01	–0·01, 0·02	< 0·00	–0·02, 0·02	0·02	< 0·00, 0·04
Proportion mediated (Stata)	–	0·13	–0·64, 0·90	−0·01	–0·3, 0·28	0·18	–2·52, 2·89	0·01	–0·20, 0·22	0·13	< 0·00, 0·26
Proportion mediated according to MacKinnon *et al.* (2000)	–	13 %		1 %		18 %		1 %		13 %	
Minimum acceptable diet											
Total causal effect	–	0·01	–0·01, 0·04	0·03	0.00, 0·05	0·07	0·04, 0·11	0·09	0·05, 0·13	0·11	0·07, 0·16
NDE	–	0·01	–0·01, 0·03	0·02	0.00, 0·05	0·07	0·04, 0·10	0·07	0·03, 0·11	0·12	0·07, 0·17
NIE	–	< 0·00	–0·02, 0·02	< 0·00	–0·02, 0·03	< 0·00	–0·02, 0·03	0·02	–0·01, 0·05	−0·01	–0·03, 0·02
Proportion mediated (Stata)	–	0·23	–5·31, 5·76	0·16	–0·54, 0·86	0·06	–0·31, 0·43	0·23	–0·04, 0·50	−0·07	–0·29, 0·15
Proportion mediated according to MacKinnon *et al.* (2000)	–	23 %		16 %		6 %		23 %		6 %	

MD, mean difference; NDE, natural direct effect; NIE, natural indirect effect; TCE, total causal effect.

All models were adjusted for age, sex (binary term: male and female) and place of residence (binary term: urban and rural).

The SEP groups are Higher-Rich: Higher mother’s education and Rich-wealth; Higher-Middle: Higher mother’s education and Middle-wealth; Higher-Poor: Higher mother’s education and Poor-wealth; Secondary or below-Rich: Secondary mother’s education and Rich-wealth; Secondary or below-Middle: Secondary or below mother’s education and Middle-wealth; Secondary or below-Poor: Secondary or below mother’s education and Poor-wealth.

MD refers to the difference in mean potential outcomes under different levels of exposure and/or mediator. It serves as the basis for estimating the TCE, the NDE and the NIE, each defined through specific contrasts of these potential outcomes. The total causal effect (TCE(k)) is a comparison between the mean potential outcome if all subjects were exposed at level k and the mean potential outcome if all subjects received the baseline level of exposure. The natural direct effect (NDE(k)) is a comparison between the mean of two potential outcomes. The first is the potential outcome if all subjects received exposure k, and subjects’ mediator(s) were set to their potential value(s) under baseline exposure. The second is the potential outcome if all subjects experienced the baseline exposure. The natural indirect effect (NIE(k)) is the difference between the TCE(k) and the NDE(k).

Following the advice of MacKinnon *et al.* (2000), the proportion mediated was calculated by dividing the absolute NIE by the sum of the NDE and NIE because suppression (when the total effect is less than the direct effect) existed in the estimated models.

The lowest SEP had a natural significant direct effect on stunting and wasting outcomes in all models. All estimated NIE were not significant. Estimated NDE of several models was greater than the TE, identified as suppression, because NIE were negative. The proportion mediated effect calculated using the summation of the absolute direct and indirect effect showed a large proportion (not statistically significant) mediated of the association between SEP and child growth outcomes (stunting and wasting) in several SEP groups.

For example, in the case of stunting (Table 3.1), the NDE of Higher-Poor SEP on stunting, expressed as an MD, was 0.07 (95 % CI = 0.03, 0.12). The NIE of FCI for the association between the Higher-Poor SEP group and stunting was < 0.00 (95 % CI = –0.02, 0.02). NDE of Higher-Poor SEP group was greater than its TE because of the negative NIE of FCI (MD = –0·01, 95 % CI = –0·3, 0·28). The proportion mediated effect calculated using the summation of absolute direct and indirect effect showed 1 % mediated of the association between SEP and stunting in the Higher-Poor SEP groups.

For example, in the case of wasting (Table 3.2), the NDE of the Higher-Poor SEP group on wasting was 0.02 (95 % CI = –0.02, 0.07). The NIE of FCI for the association between the Higher-Poor SEP group and wasting was 0.00 (95 % CI = –0.01, 0.02). The proportion mediated effect showed 17 % mediated of the association between SEP and wasting in the Higher-Poor SEP groups.

**Table 3.2:** Counterfactual mediation models testing the extent to which the association of socio-economic position with wasting is mediated by family care index and, separately, minimum acceptable diet

	Exposure										
		Higher-Middle		Higher-Poor		Secondary or below-Rich		Secondary or below-Middle		Secondary or below-Poor	
	Higher-Rich (referent)	MD	95 % CI	MD	95 % CI	MD	95 % CI	MD	95 % CI	MD	95 % CI
Mediator											
Family care index											
Total causal effect	–	0·01	–0·03, 0·04	0·03	–0·01, 0·07	0·01	–0·03, 0·06	0·03	0.00, 0·07	0·05	0·02, 0·09
Natural direct effect	–	< 0·00	–0·03, 0·03	0·02	–0·02, 0·07	0·00	–0·04, 0·04	0·03	–0·01, 0·06	0·05	0·02, 0·09
Natural indirect effect	–	0·01	–0·01, 0·02	0	–0·01, 0·02	0·01	0.00, 0·03	0.00	–0·01, 0·02	< 0·00	–0·02, 0·02
Proportion mediated (Stata)	–	0·88	–5·52, 7·28	0·17	–2·61, 2·94	1.00	–4·2, 6·2	0·11	–3·42, 3·64	< 0·00	–0·33, 0·33
Proportion mediated according to MacKinnon *et al.* (2000)	–	88 %		17 %		100 %		11 %		0 %	
Minimum acceptable diet											
Total causal effect	–	< 0·00	–0·05, 0·05	0·01	–0·05, 0·07	0·04	–0·03, 0·11	0·03	–0·02, 0·08	0·06	0·01, 0·10
Natural direct effect	–	−0·01	–0·05, 0·04	0·03	–0·03, 0·09	0·03	–0·04, 0·10	0·02	–0·03, 0·07	0·06	0·01, 0·11
Natural indirect effect	–	0·01	–0·01, 0·03	−0·02	–0·04, 0.00	0·01	–0·01, 0·03	0·01	–0·02, 0·03	−0·01	–0·03, 0·02
Proportion mediated (Stata)	–	3·14	–3·56, 9·85	−1·32	–5·35, 2·71	0·22	–5·07, 5·51	0·25	–4·91, 5·42	−0·09	–1·16, 0·98
Proportion mediated according to MacKinnon *et al.* (2000)	–	59 %		36 %		22 %		25 %		8 %	

MD, mean difference; SEP, socio-economic position.

See Table 3.1 note for definitions of SEP groups and causal effect measures.

All models were adjusted for age, sex (binary term: male and female) and place of residence (binary term: urban and rural).

The results did not show a clear pattern of the proportion mediated by FCI and MAD across the SEP categories. For example, our study found 13 % of the association between stunting and Higher-Middle group (compared to Higher-Rich group) mediated by FCI. The same proportion was mediated by FCI in the Secondary or below-Poor SEP group.

The proportion of the association between SEP and stunting mediated by FCI ranged from 13 % to 18 % in the Higher-Middle, Secondary or below-Rich, and Secondary or below-Poor SEP groups. It was only 1 % in the Higher-Poor and Secondary or below-Middle groups. Mediation by MAD ranged from 16 % to 23 % in the Higher-Middle, Higher-Poor and Secondary or below-Middle groups, but was lower (6 %) in the Secondary or below-Rich and Secondary or below-Poor groups.

For the association between SEP and wasting, the proportion mediated by FCI and MAD ranged from 11 % to 100 % across all SEP groups, except in the Secondary or below-Poor group, where mediation was 0 % for FCI and 8 % for MAD.

## Discussion

The key finding of this study is the absence of a clear pattern in the proportion of the association between SEP and child undernutrition that was mediated by FCI and MAD across different SEP categories. We found that the composite SEP variable of mother’s education and wealth index was strongly associated with the prevalence of stunting and wasting in Sri Lanka. The mediated proportion of the association between SEP and stunting and wasting by MAD or FCI was not statistically significant. Although not statistically significant, a substantial proportion of the association between SEP and stunting or wasting was mediated by FCI and MAD in specific subgroups. For stunting, mediation by FCI and MAD ranged from 13 % to 23 % in several SEP combinations (Higher mother’s education and Middle-wealth group; Secondary or below mother’s education and Rich-wealth group; Secondary or below mother’s education and Poor-wealth group; and Secondary or below mother’s education and Middle-wealth group). In contrast, mediation of wasting showed wide variation (11 % to 100 %), with minimal mediation observed in the most disadvantaged subgroup, suggesting potential inequities in the pathways leading to undernutrition.

Our finding was not similar to the study of Dwomoh *et al.* (2022)^(11)^ which investigated the extent to which associations of mother’s education and household wealth with stunting and wasting were mediated by MAD, MMF and MDD in West Africa. They investigated a large sample of mothers (*n* 132 448) with children under-5 in thirteen low-and middle-income countries. Dwomoh *et al.*’s survey design and data collection methods were similar to our study, as both studies analysed DHS data. They reported a higher proportion of the association between mother’s education (35·9 %, 95 % CI = 12·4, 59·3) and household wealth (37 %, 95 % CI = 11·5, 61·5) with stunting mediated by MAD, compared to our study. This was more than twice the proportion we found: only 12 % of the association between SEP (measured by mother’s education and household wealth) and stunting was mediated by MAD (Table 3.3). This may be due to the stronger association between child diet (MAD) and stunting in West African countries than in Sri Lanka. However, the comparison between Dwonmoh *et al.* and our study was limited because the models used in the two studies are not the same. They employed a generalised structural equation model approach, which estimates associations based on model structure, while we used counterfactual mediation analysis via G-computation, which allows for causal interpretation under specific assumptions. These differing approaches yield estimates that are not directly comparable. The descriptive statistics from Dwomoh *et al.*’s (2022) study indicate patterns that suggest a possible association between stunting and SEP. The percentage of stunted children in the not meeting MAD group in their study was 35 %. In our study, it was only 20 % (Table 2). The reason for the lack of significance in mediation proportions in our study compared to Dwomoh *et al.*’s study is likely due to the statistical power of a large sample. The relatively small sample size in our study may not have reflected the actual mediation effect of diet and household environment in sub-populations because studies with smaller sample sizes have less ability to detect real effects, and the results are more uncertain.

**Table 3.3:** Counterfactual mediation models testing the extent to which the association of socio-economic position with stunting (and wasting) is mediated by family care index and, separately, minimum acceptable diet. In these models, the exposure variable (SEP) was included as a single combined variable, rather than broken down into its specific levels, Higher-Rich, Higher-Middle, Higher-Poor, Secondary or below-Rich, Secondary or below-Middle and Secondary or below-Poor

	Stunting		Wasting	
	MD	95 % CI	MD	95 % CI
FCI				
Total effect	0·07	0·01, 6·01	0·02	0·01, 1·52
Natural direct effect	0·08	0·01, 6·70	0·03	0·01, 2·57
Natural indirect effect	−0·01	0·01, –1·03	−0·01	0·01, –1·7
Proportion mediated (Stata)	−0·12	0·12, –1·04	−0·73	1·50, –0·48
Proportion mediated % (MacKinnon *et al.*)	10 %		30 %	
MAD				
Total effect	0·07	0·01, 4·77	0·04	0·02, 2·76
Natural direct effect	0·08	0·01, 5·61	0·05	0·02, 3·61
Natural indirect effect (via MAD)	−0·01	0·01, –0·89	−0·01	0·01, –1·17
Proportion mediated (Stata)	−0·15	0·23, –0·67	−0·30	1·06, –0·29
Proportion mediated % (MacKinnon *et al.*)	12 %		19 %	

SEP, socio-economic position; MD, mean difference; FCI, family care indicator; MAD, minimum acceptable diet.

See Table 3.1 note for definitions of causal effect measures.

All models were adjusted for age, sex (binary term: male and female) and place of residence (binary term: urban and rural).

In Sri Lanka, we found that although the mediation of the association between SEP and wasting by MAD was inconsistent and not statistically significant, the proportion mediated was relatively large in some SEP groups. This may be due to dietary indicators being strong predictors of wasting in Sri Lanka^(21,22)^. For example, Aboagye *et al.* 2021 and Eini-Zinab *et al.* 2021^(22,23)^ reported that diet is strongly associated with both SEP and wasting in infants and young children in low-and middle-income countries. Since children’s diets begin to change after 6 months of age with the ending of exclusive breast-feeding, SEP may influence wasting through its impact on dietary quality, such as MAD, which could act as a mediator on this association^(11,24)^.

There was not a clear pattern in the proportion mediated of the association of SEP with stunting or wasting by MAD or FCI. The reason for this inconsistent trend in the proportion of mediation of MAD and FCI across the SEP groups may be due to the effect of other mediators such as water supply, sanitation and hygiene. For example, Raihan *et al.* (2017)^(25)^ reported a 60 % mediation effect likely to be from water supply, sanitation and hygiene (WASH) on the association of socio-economic variables with weight-for-height z-score among under-5 children in Bangladesh.

We found suppression due to negative NIE through MAD and FCI. The negative indirect effect can occur due to either the association between SEP and the mediator being negative or the association between mediator and stunting being negative. In our study, we found no significant association (unadjusted) between mediators and outcomes for FCI and wasting, and MAD with both stunting and wasting. There was a significant association between FCI and stunting. However, the association between SEP and mediators was significant. This may be the reason for the negative NIE we found. Therefore, we manually calculated the proportions of the effect (proportion mediations) that was explained by each mediator, MAD and FCI, using NIE and NDE without considering their positive or negative signs to show how much of the association between SEP and child growth outcomes (stunting and wasting) could be mediated by MAD and FCI in this study.

### Limitation of the study

Although not all five domains of the UNICEF Nurturing Care Framework could be represented due to DHS data limitations, our choice of MAD and FCI was directly informed by the framework, ensuring that the study remains conceptually anchored in this model of early child development. Several important variables related to child undernutrition were not directly included in our analysis. Water and sanitation variables were not analysed separately because they are already incorporated into the DHS Wealth Index, which we used to measure SEP. In addition, 151 children (3 %) had recent episodes of diarrhoea, and although they were included in the dataset, this variable was not adjusted for in the analysis. As acute illness can temporarily affect anthropometric measurements, this may have introduced a small degree of measurement bias. Furthermore, FCI data were self-reported, which may introduce social desirability bias. These factors should be considered when interpreting the findings.

The strength of this research is the use of a nationally representative sample consistently collected using DHS measures in Sri Lanka to facilitate a coherent analysis. The data were collected in 2016, and although they remain the most comprehensive nationally representative dataset available, the current situation may have changed. However, the associations examined are unlikely to differ substantially over time and thus remain informative for understanding mechanisms. Counterfactual data analysis using the g-computation procedure (‘gformula’) is another study strength. This method allows the estimation of the natural direct and indirect effects adjusted for confounders. We only adjusted for age, sex and place of residence because they are biological (age and sex) and non-biological factors which confound the association of the mediator with the outcome, the exposure with the outcome and the exposure with the mediator. However, other confounding variables (e.g. health service and infections) that could potentially confound the relationship we studied were not included because the data for these variables were not included in the Demographic and Health Surveys (DHS-2016) data of Sri Lanka. The analysis was also constrained to the SEP indicators collected in the DHS surveys.

### Conclusion

Our results did not find consistent or strong evidence that the associations of SEP with childhood stunting and wasting in Sri Lanka are mediated by MAD and FCI. Further studies with larger sample sizes are needed in order to more precisely estimate the proportions mediated of the association between SEP and stunting and wasting by child nutrition and household care environment in Sri Lanka.

## Supporting information

Chandrasenage et al. supplementary material Chandrasenage et al. supplementary material

## Data Availability

Not applicable

## Supplementary material

For supplementary material accompanying this paper visit https://doi.org/10.1017/S1368980026101888
