# Supplementary material for: Do minimum acceptable diet and family caregiving mediate the associations of maternal education and household wealth with childhood stunting and wasting in Sri Lanka?
Source: Public Health Nutr. 2026 Jan 23;29(1):e34. doi: 10.1017/S1368980026101888 (PMC12917429; doi:10.1017/S1368980026101888)
Supplement: Chandrasenage et al. supplementary material [file S1368980026101888sup001.docx]

Supplementary materials

Table S1. Separately estimated associations between exposure and outcomes, exposure and mediators, and mediators and outcomes.

|  | **FCI Sample (N= 4325)** | | |  | **MAD sample (N= 2190)** | | |
| --- | --- | --- | --- | --- | --- | --- | --- |
| **SEP** | **FCI** | **Stunting** | **Wasting** |  | **MAD** | **Stunting** | **Wasting** |
|  |  | **OR (95% CI)** |  |  |  | **OR (95% CI)** |  |
| Higher & Rich (referent) | - | - | - |  | - | - | - |
| Higher & Middle | 1.33 (1.08, 1.64) | 1.51 (1.16, 1.96) | 1.06 (0.80, 1.41) |  | 1.42 (1.09, 1.87) | 1.85 (1.29, 2.66) | 1.15 (0.77, 1.71) |
| Higher & Poor | 2.06 (1.58, 2.67) | 1.75 (1.29, 2.36) | 1.33 (0.96, 1.85) |  | 2.00 (1.45, 2.76) | 1.61 (1.04, 2.50) | 1.47 (0.93, 2.31) |
| Secondary-or-below & Rich | 2.26 (1.70, 3.02) | 1.43 (1.01, 2.02) | 1.11 (0.76, 1.63) |  | 1.05 (0.70, 1.55) | 1.63 (0.98, 2.72) | 1.35 (0.79, 2.31) |
| Secondary-or-below & Middle | 2.53 (2.03, 3.14) | 1.98 (1.54, 2.55) | 1.38 (1.05, 1.81) |  | 1.19 (0.90, 1.56) | 2.24 (1.58, 3.19) | 1.47 (1.00, 2.16) |
| Secondary-or-below & Poor | 3.70 (3.03, 4.52) | 2.78 (2.22, 3.47) | 1.69 (1.33, 2.15) |  | 2.05 (1.60, 2.63) | 2.89 (2.10, 3.99) | 1.85 (1.31, 2.60) |
| **FCI or** |  |  |  |  |  |  |  |
| Adequate (referent) | -- | -- | -- |  | -- | -- | -- |
| Inadequate |  | 1.47 (1.24, 1.74) | 1.14 (0.94, 1.38) |  | -- | -- | -- |
| **MAD** |  |  |  |  |  |  |  |
| Adequate (referent) | -- | -- | -- |  | -- | -- | -- |
| Inadequate |  |  |  |  |  | 0.99 (0.79, 1.23) | 1.00 (0.78, 1.28) |

All models adjusted for age, sex and resident place
